# Spatio-Temporal Changes in Land Use and Habitat Quality of Hobq Desert along the Yellow River Section

**DOI:** 10.3390/ijerph20043599

**Published:** 2023-02-17

**Authors:** Ruibing Meng, Jiale Cai, Hui Xin, Zhongju Meng, Xiaohong Dang, Yanlong Han

**Affiliations:** College of Desert Control Science and Engineering, Inner Mongolia Agricultural University, Hohhot 010010, China

**Keywords:** land-use change, habit quality, InVEST model, PLUS model, Hobq Desert

## Abstract

As a key area in the Yellow River basin for sand control and management, the land change process in the Hobq Desert plays a crucial role in keeping the river and desert ecosystems and promoting the construction of ecological civilization in human systems. Based on multi-temporal remote sensing from 1991 to 2019 in the Hobq Desert along the Yellow River section, this study selected spatial statistical methods (land-use monitoring and landscape metrics) to examine land-use change dynamics. Then, we evaluated habitat quality using the InVEST model and quantitatively analyzed the factors causing spatial changes in habitat quality using geographic detectors. Finally, this paper predicted the pattern of land use and habitat quality in 2030 using the PLUS model. The results reveal that (1) from 1991 to 2019, the total area of forest grassland increased by 3572.5 km^2^, providing the most vegetation cover, and the sandy land and water area decreased continuously, while the cultivated land and construction land increased. There were 38.01% conversions of land types, with the land-use dynamic decreasing the greatest in sandy land (−12.66%) and increasing the greatest in construction land (9.26%); the comprehensive land-use dynamics were the highest in 2010–2019 (1.68%), which was the most active stage during our study period. (2) Both of the landscape indices NP and PD showed “N” type fluctuations during 1991–2019, and CONTAG and LSI rose from 69.19% to 70.29% and 36.01% to 38.89%, respectively, indicating that the land-use degree of landscape fragmentation increased, landscape connectivity turned better, and landscape dominance was enhanced, balanced, and developed evenly in overall landscape type. (3) From the overall region analysis, the average habitat quality in 1991, 2000, 2010, and 2019 was 0.3565, 0.5108, 0.5879, and 0.6482, respectively, with the overall habitat value showing a gradually increasing trend. Spatially, the habitat quality along the Yellow River section of the Hobq Desert has a certain regularity, and the overall pattern there is high in the south and low in the north, high in the east and west, and low in the middle. (4) The change in land use between 2019 and 2030 is similar to the previous period, but the change rate is generally lower. The habitat quality improved significantly, with the growth of high and medium habitat quality.

## 1. Introduction

Land plays a core role in the compound system of “population—resources—environment—development (PRED)” [1,2]. The change in land use results from human activities changing the surface of Earth and natural ecosystems, which also reveal the spatio-temporal dynamic process of the landscape in Earth’s surface layer [3]. Many ecologically fragile regions face the trade-off between economic development and ecological protection. The rapid development of urbanization has uncertain impacts on ecological environment in arid areas, resulting in several problems, such as reducing the quantity and quality of land resources and declining groundwater levels [4,5,6]. The situation intensifies the mutual transformation among various land-use types and restricts the region’s sustainability. On the one hand, corresponding changes in the desert landscape will occur and the surrounding land will follow the influence of natural factors [7]. On the other hand, various social activities of human beings have changed land-use patterns and development intensity to varying degrees, and the spatial use within the desert and the land-use types at the edge of the desert are constantly disintegrated and reconstructed, which have caused fragmentation, degradation, and even loss of regional habitat, resulting in serious challenges to it [8,9]. Therefore, it is important for ecologically fragile areas to evaluate and investigate the land-use change characteristics, as they can assist people to formulate policies and achieve the target of protecting the ecosystem [10,11].

Monitoring habitat quality requires the integration of diverse characteristics of ecosystems. Early habitat quality was evaluated mainly based on field-measured habitat indicators for small-scale habitats, such as vegetation succession, soil components, and aquatic organisms [12,13,14]. Habitat index modeling has high evaluation accuracy and pertinence, but the number of samples is limited and time-consuming [15]. With the development of geographic information technology, the integration technology based on RS and GIS makes the research more quantitative and visual [16]. Ecological models include the Mexent model, habitat suitability index (HIS) mode, and land integrated valuation of ecosystem services and tradeoffs (InVEST) [17,18,19,20]. Among them, the InVEST model, developed by the Natural Capital Project of the United States, has the characteristics of simple operation and easy access, and has been extensively applied to spatial planning, ecological compensation, risk management, and other environmental management decisions in more than 20 countries and regions [21,22,23,24]. For example, Imran et al. [21] evaluated the carbon stock of forest types in mountainous areas and carried out mapping analysis on the basis of the InVEST model. Mdk et al. [22] investigated habitat quality changes based on land use in Ghana and the Ivory Coast over time, and presented a general overview of habitat quality in these two countries. Terrado et al. [23] assessed habitat quality in the Italian watershed on the basis of the InVEST model.

The above studies are a critical guideline for advancing habitat quality research and they have carried out numerous ecosystem service assessments for a wide range of areas, such as urban agglomeration systems, mountain forest lands, watershed units, etc. They have contributed to the advancement of habitat quality assessments, but were limited by the lack of access to land-use data in history or for future scenarios [25,26]. Thus, it is increasingly necessary to explore the development of habitat quality from the perspective of simulation, which could bring lessons to ecosystem protection planning and habitat quality management. Predicting land use can be experimented on using a variety of models [27,28]. The study used a patch-generating land-use simulation model (PLUS) integrated with a Markov chain to predict land use in the future. Compared to Logistic-CA, CLUE-S, FLUS, and other models, the PLUS model avoids the defect of exponential growth of transformation types with the increase in categories [29]. The PLUS model retains the ability to mine the driving mechanism of land-use change in a certain period, which can effectively simulate the complex evolution process of multiple land types [30].

From what has been discussed above, two models have been applied widely and effectively, but there have only been a few studies on the coupled InVEST-PLUS model to assess and predict the spatio-temporal change pattern of land use and that employed geographic detectors to explore the spatial heterogeneity of habitat quality. Combining land-use change with habitat quality can be better measured from the spatial and temporal dimensions, thus realizing a multi-model coupled correlation analysis. More importantly, this can capture the land-use patch-level changes more accurately than a single model. Therefore, the realization of multi-model coupled correlation analysis is significant for developing land-use change simulation studies and establishing the basis for in-depth analysis of spatio-temporal variation characteristics in habitat quality.

Hobq Desert control has embarked on a path with Chinese characteristics, providing a method studied in China for global sustainable development [31]. At present, the northwest arid area is witnessing rapid development with land use in a transitional state, coupled with the increasingly serious problems of the Yellow River basin in recent years, particularly water shortage, the weakening of resources, and environmental carrying capacity [32,33]. At the same time, Hangjin Banner, the county with the longest flow in the Yellow River basin, is suffering from serious wind erosion and desertification. It has been facing the dual pressures of economic growth and ecological restoration in recent years, with frequent land-use-type conversion [34]. In this way, ecological environmental protection has become extremely necessary for sustainable and high-quality development in this area.

Due to this, we selected Hangjin Banner, which sits on the middle and upper reaches of the Hobq Desert along the Yellow River, as a typical research area. This study relies on spatial quantification tools for habitat quality and land use simulation techniques to reveal the previous and future changes in land use and habitat quality, combined with a geographical detector to explore the drivers of spatial variation affecting habitat quality. It is not only useful for a deeper perspective on the previous change characteristics and future growth patterns of ecologically fragile areas along the Yellow River section of the Hobq Desert, but it also facilitates different planning proposals for policy-makers and specific stakeholders (e.g., farmers and ranchers). The research objectives of this paper were as follows: (1) via land-use dynamic indicators, the land-use transfer matrix, and the landscape pattern index, we monitored and analyzed the long-term changes in land use in 1991, 2000, 2010, and 2019. (2) We predicted the new situation of land use and habitat quality in 2030 using the PLUS model. (3) Using the InVEST model to quantify the habitat quality status from 1991 to 2030, we superimposed it to analyze the spatial variation regularity with different habitat quality classes, and combined this with the geographical detector to explore the impact caused by different drivers on habitat quality spatial variation.

## 2. Materials and Methods

### 2.1. Study Area

**Figure 1:**
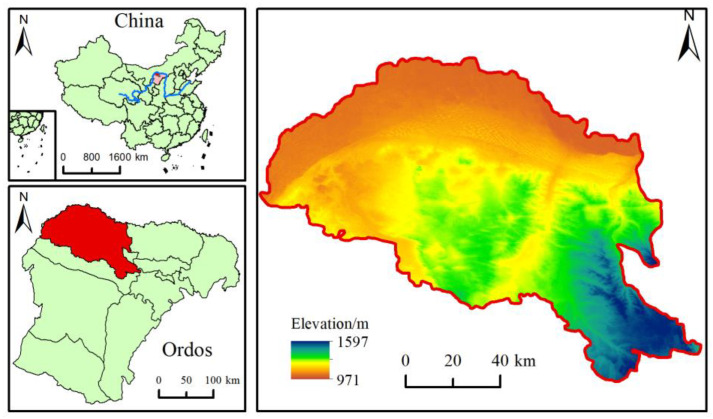
Location along the Yellow River section of the Hobq Desert.

The Hobq Desert along the Yellow River section (39°54′ N–40°87′ N, 107°11′ E–109°30′ E) is located in the northwestern part of the Ordos Plateau (Figure 1). West and north are bounded by the Yellow River, its main water source, along with the tributaries of rivers such as Maubulakontou and Bursetagou. The topography of the territory has an obvious band distribution pattern, with high southeast and low northwest terrain. The terrain is dominated by fixed and semi-fixed sands and fluvial sands. Soils are mainly wind–sand and brown calcium soils, which are fragile and vulnerable. The climate is in a temperate semiarid continental climate zone, with a large temperature difference between daytime and nighttime, an average annual temperature of 6.8 °C, an average annual evaporation of 2720 mm, and an average annual precipitation of 245 mm. In 2019, the total population of Hangjin Banner was 142,800 people, GDP reached USD 1.82 billion, and the per capita disposable income of farmers and herders reached USD 2903 [35].

### 2.2. Data Sources and Data Processing

The data sources were based on land-use data of 30 m resolution from 1991, 2000, 2010, and 2019 in Hangjin Banner from the Geospatial Data Cloud (https://www.gscloud.cn/; accessed on 11 October 2020) [36]. Where the original projection coordinate system in 1991 was WGS_1984_UTM_Zone_48N, we changed it to the same projection coordinate system as for 2000, 2010, and 2019; therefore, this was an area error in the projection transformation (<1 km^2^). According to the classification standard of the Land-Use Status Classification (GB/T210102017), we classified land-use types into cultivated land, forest grassland, water area, construction land, unused land, and sandy land. After radiometric calibration and atmospheric correction pre-processing, image information was extracted from the study area using supervised classification.

According to the accuracy and realism of the PLUS model, we selected 12 drivers affecting land-use change from natural, socio-economic, and accessibility aspects to carry out land-use prediction research. DEM data were derived from the Geospatial Data Cloud, and slope was calculated using ArcGIS10.8’s slope module. Meteorological data (annual average temperature and annual precipitation) were taken from the National Meteorological Science Data Center (http://www.geodata.cn/; accessed on 30 October 2022) [37]. Soil type and erosion, water system distribution, and socio-economic data (GDP and population density) were taken from the Data Center for Resource and Environmental Sciences, Chinese Academy of Sciences (https://www.resdc.cn; accessed on 30 October 2022) [38]. The distribution of current roads, highways, railroads, and settlements was derived from the Global Geographic Information Resources Directory Service (https://www.webmap.cn/; accessed on 28 October 2022) [39], and the accessibility factor was calculated using Euclidean distance tools of ArcGIS 10.8. All data were transferred to 30 × 30 m raster files with the same rows and columns according to PLUS model input data requirements.

### 2.3. Research Methods

#### 2.3.1. Land-Use Monitoring

(1) Dynamic indicators of land use

Land-use dynamics are based on the statistical data of land use, which can describe the spatial distribution of land use in a study area over a specified time period [40]. Generally, they include the land-use dynamic attitude (*K*) and comprehensive land-use dynamic attitude (*LC*).

Land-use dynamic attitude (*K*) describes the speeds of change in certain land-use types within a particular time of the study area. We used it to calculate the land change in each type along the Yellow River section of the Hobq Desert during different periods from 1991 to 2019. The mathematical expression is as follows:

(1) $$K=\frac{U_{b}-U_{a}}{U_{a}}\times \frac{1}{T}\times 100\%$$

where *K* is the dynamic degree of certain land-use types during the study period; *U_a_* and *U_b_* are the areas of particular land-use type at the start and the end of the study, respectively; and *T* is the length of the study period.

Namely, comprehensive land-use dynamic attitude (*LC*) indicates the degree of change in the study area’s overall pattern of all land-use types. It is able to express the integral situation of land-use change in a particular period. The formula is:

(2) $$LC=\left(\frac{\sum_{i=1}^{n}\Delta LU(ij)}{2\sum_{i=1}^{n}LUi}\right)\times \frac{1}{T}\times 100\%$$

In the formula, *LC* is the comprehensive land-use dynamic; *LU_i_* is the starting area of Class *i*; Δ*LU_(ij)_* is the absolute value of land-use-type conversion area at study time; and *T* is research time.

(2) Land-use transfer change

The Markov transfer matrix is a statistical method used to indicate the conversion area between different land-use types quantitatively. Moreover, the land-use transfer matrix reveals the process by which various land types transform into each other at specific time intervals. We used the spatial overlay analysis of GIS software to obtain the dynamic transfer change in different periods. This research was primarily divided into four periods: from 1991 to 2000, from 2000 to 2010, from 2010 to 2019, and from 1991 to 2019. The mathematical expression of the transfer matrix is as follows [41]:

(3) $$P_{mn}=\left[\begin{array}{cccc} P_{11} & P_{12} & \ldots & P_{1n}\\ P_{21} & P_{22} & \ldots & P_{2n}\\ \ldots & \ldots & \ldots & \ldots \\ P_{n1} & P_{n2} & \ldots & P_{nn} \end{array}\right]$$

where *P_mn_* expresses the transfer probability of land-use type from class m to class n, and m and n represent land-use types in the Hobq Desert in the initial and last study stages, respectively.

#### 2.3.2. Description Index of Landscape Pattern

Landscape pattern refers to the spatial design made by a series of landscape elements of diverse sizes and shapes arranged in different ways in the space, including the number, type, and spatial distribution and configuration of landscape components, which have an important influence on the function of regional ecosystems [42]. Considering the different ecological significance of landscape indicators and the large correlation between certain indicators, we selected the patch density (PD), number of patches (NP), landscape shape index (LSI), largest patch index (LPI), splitting index (SPLIT), Shannon’s diversity index (SHDI), aggregation index (AI), and contagion index (CONTAG) to monitor landscape pattern information, which was calculated using fragstats 4.2. The expressions and ecological meaning are shown in Table 1.

#### 2.3.3. Assessment of Habitat Quality

We used the easily and highly visualized InVEST 3.12.0 software habitat quality module to assess spatio-temporal evolution characteristics of the Hobq Desert along the Yellow River section from 1991 to 2030. The habitat quality module mainly uses the relative impact of each threat, the relative sensitivity of each habitat type to each threat, the distance between the habitat raster and the threat source, and the information on land use cover and biodiversity threat factors to generate a habitat quality map [22,43]. When habitat quality is excellent, resources and conditions are met and biodiversity development is safeguarded, while areas with lower habitat quality indicate reduced sustainability, elasticity, and self-restoration capacity [44].

Habitat degradation indicates the degree of degradation following the impact of threat factors and the model suggests two functions to describe the impact of the threats over space: linear decay and exponential decay (Equations (5) and (6)). The formulas are as follows:

(4) $$D_{xj}=\sum_{1}^{r}\sum_{1}^{y}\left(\frac{w_{r}}{\sum_{r=1}^{n}w_{r}}\right)r_{y}i_{rxy}\beta_{x}S_{jr}$$

(5) $$i_{rxy}=1-\left(\frac{d_{xy}}{d_{r\max}}\right)\left(\mathrm{Linear}\;\mathrm{decay}\right)$$

(6) $$i_{rxy}=\exp\left[-\left(\frac{2.99}{d_{r\max}}\right)d_{xy}\right]\left(\mathrm{Exponential}\;\mathrm{decay}\right)$$

where *D_xj_* is the degree of habitat degradation at spatial unit *x*; *r* is habitat threat driver; *y* is the grid responding to the threat driver *r*; *w_r_* is the weight of habitat threat factor; *r_y_* is the stress value of the threat factor; *β_x_* is the protected level of the habitat; *i_rxy_* is the effect of the threat factor *r* in raster y on raster *x*; *S_jr_* is the sensitivity of habitat j to the stress factor *r*; *d_xy_* is the linear distance between the spatial unit *x* and *j*; and *d_rmax_* is the maximum stress distance of the threat factor.

Habitat quality is a dimensionless measure for evaluating the quality of regional habitats, with a value between 0 and 1; the higher the value, the better the habitat. The calculation is:

(7) $$Q_{xj}=H_{xj}\left[1-\left(\frac{D_{xj}^{2}}{D_{xj}^{z}+k^{z}}\right)\right]$$

where *Q_xj_* is habitat quality of land class *k* at spatial unit *i*; *H_xj_* is habitat suitability for habitat type *j*; *k* is the half-saturation parameter; $D_{xj}^{z}$ is the degree of habitat degradation of spatial unit *x*; and *z* is the default model parameter.

Based on the situation along the Yellow River section of the Hobq Desert, cultivated land, construction land, unused land, and sandy land with high intensity of human disturbance are threat factors to habitat quality. Referring to the InVEST model instruction manual and related research results, the weights, maximum influence distances, and spatial decline types were set (Table 2), and the habitat matters of each species to the threat factors were set (Table 3).

#### 2.3.4. Prediction Based on PLUS Model

**Figure 2:**
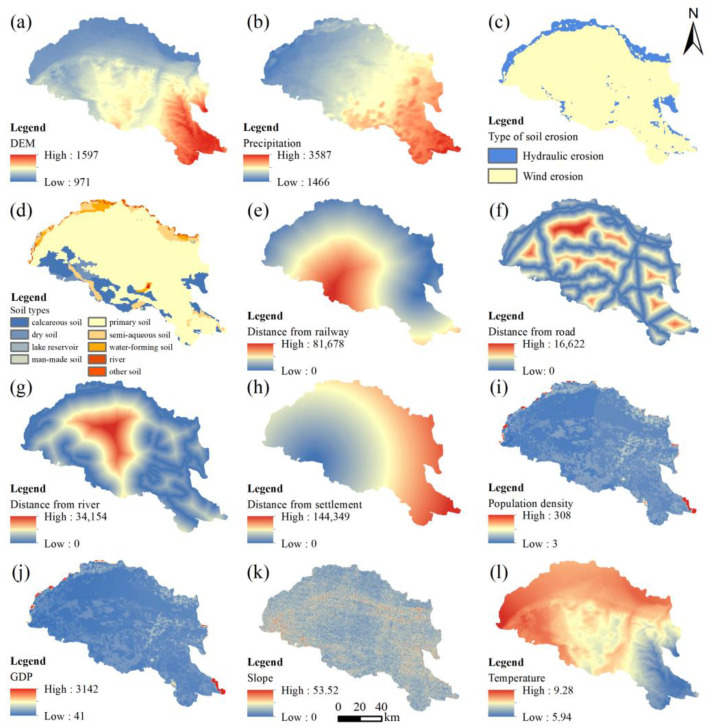
Land-use driving factors. (**a**) DEM. (**b**) Precipitation. (**c**) Type of soil erosion. (**d**) Soil types. (**e**) Distance from railway. (**f**) Distance from road. (**g**) Distance from river. (**h**) Distance from settlement. (**i**) Population density. (**j**) GDP. (**k**) Slope. (**l**) Temperature.

The PLUS model simulates land-use patterns with higher simulation accuracy and a more authentic measure of the landscape [31]. The model consists of transformational rule strategies based on a rule mining approach for land expansion analysis and a CA model based on a multi-type stochastic seed mechanism [45]. First, based on the actual situation in the study area and data availability, we selected 12 factors, including elevation, slope, temperature, precipitation, GDP, population density, distance to settlements, and distance to rivers, etc., as shown in Figure 2. Secondly, the development probability of each landscape type was calculated using the land expansion analysis strategy (LEAS) module. At last, we combined relevant parameters such as the target number of image units, the probability of random patch seeding, and the neighborhood factor for each type of future land use to realize the simulation of landscape-type change in the study area. The kappa and FOM coefficient were chosen to estimate the reliability of the simulation results, and higher values indicate higher accuracy.

#### 2.3.5. Geographical Detector Method

The geographic detector is a statistical method that detects spatial heterogeneity and analyzes the interrelationship between drivers [46]. It is based on the principle that divides the research area into several subareas. In the case of spatial heterogeneity, the sum of variances of the sub-regions is less than the total variance of the site. In the case of statistical correlation, the spatial distributions of the two variables tend to be similar. The geodetector includes 4 aspects of detection: factor detector, ecological detector, risk detector, and interaction detector. In this study, we used factor detection and interaction detection to investigate the factors influencing the spatial evolution characteristics of habitat quality in the Hobq Desert. The mathematical expression of the geographic detector is:

(8) $$q=1-\frac{\sum_{h=1}^{L}N_{h}\sigma_{h}^{2}}{N\sigma^{2}}$$

where *q* can reflect the explanatory power of factors on spatial variation in habitat quality; *N_h_* and *N* are the total grid unit numbers of layer *h* and the whole area, respectively; *σ_h_^2^* and *σ*^2^ denote the *Y*-value variance of layer *h* and the overall in this study, respectively; and *L* is the classification of variable *Y* or factor *X*. By testing the *q* value with *F* statistics, the larger the value, the greater the explanatory power.

The spatial heterogeneity of habitat quality is formed under the action of various factors. In this paper, we examined the basic characteristics and development status of the desert in a comprehensive manner and selected natural factors (temperature, precipitation, slope, NDVI, dem, soil types), social factors (population density, GDP), and LUCC to quantitatively analyze the impact of each driver on landscape habitat quality along the Yellow River section of the Hobq Desert. By applying the natural breakpoint method, the grade factors other than land-use type and soil type were split into 7 grades.

## 3. Results

### 3.1. Analysis of Land-Use Change from 1991 to 2019

#### 3.1.1. Land-Use Type Change

We used statistics to collect land-use data along the Yellow River section of the Hobq Desert in 1991, 2000, 2010, and 2019 (Table 4). The sandy land was the predominant land-use type in the initial period, followed by forest grassland, cultivated land, and water area, and relatively little unused and construction land. In 2000, environmental greening promoted the development of massive quantities of forest grassland on sandy land, transforming the land-use type to mainly forest grassland. The water area changed instability, and the cultivated land expanded rapidly. Sandy land, water area, and cultivated land areas all experienced −53.26%, −44.71%, and 79.78% changes, respectively.

**Figure 3:**
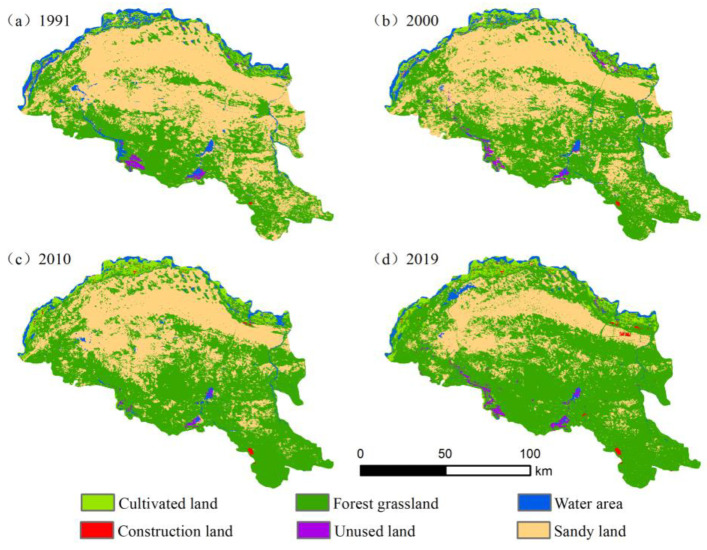
Spatial variation in land use in 1991, 2000, 2010, and 2019.

We can see that the cultivated land is concentrated in the distribution changes along the banks of the Yellow River (Figure 3). The area has a rising trend over time, with a total increase of 227.03 km^2^. The construction land was located in the desert edge and relatively gentle landscape areas, the site increased about six times, and the rate of expansion was relatively slow. Sandy land was concentrated in the south-central part of Hangjin Banner, and the cultivated land’s edge changed significantly. Unused land areas show a decrease first and an increase later, which is mainly saline land distributed near the water. The water area is distributed primarily on strips by the Yellow River on the northwest border of the desert, and there are also some seasonal rivers and lakes spread within, with a total area reduction of 260.82 km^2^. Overall, these results indicate that land-use changes spatial intensive distribution, the main body is more stable, and the region is dynamic. The distribution of vegetation cover extends from outside to inside and from south to north.

#### 3.1.2. Land-Use Dynamic Change

What is striking in Table 5 is the dramatic decline in sandy land during the entire study period, followed by water area; the land-use dynamics were −12.66% and −8.98%, respectively. From 1991 to 2000, there was less change by land type than the rapid development of construction land in 2000–2010, with the land-use dynamic being as much as 13.89%. From 2010 to 2019, the unused land expanded sharply, with a land-use dynamic of 25.55%. This indicates that the region has been subjected to remediation and the development of sandy land. This has led to a rapid increase in construction and unused land but a drastic decrease in water areas due to poor development management. In summary, the percentage of comprehensive land-use dynamics is 0.68%. Among them, the comprehensive land-use dynamics in the early and middle periods were 1.22% and 1.08%, respectively, with moderate land-use changes. The composite index for the latter period was 1.69%, with dynamic changes in various categories and intensive contradictions between humankind and nature.

#### 3.1.3. Land-Use Transfer Matrix

**Figure 4:**
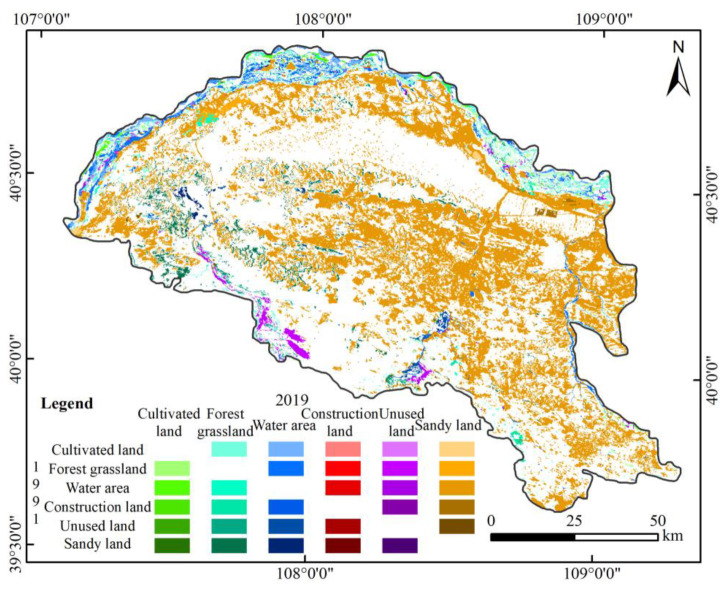
The land-use transfer changes along the Yellow River section of the Hobq Desert from 1991 to 2019.

During 1991–2019, the Yellow River section of the Hobq Desert experienced frequent land conversions, in which 31.80% of the land was transformed (Figure 4). Three transfer directions were evident: sandy land transfers out, water area transfers out, and forest grassland transfers in. Of these, the transformation of sandy land occurred in the greatest area, covering 3729.06 km^2^, which accounts for 90.96% of the total transformed forest grassland. The reduction in water area is also more obvious, and it is mainly turned to cultivated land. From these, one can see that the implementation of environmental policies in the Hobq Desert during the study period was effective, and the sandy ground was effectively managed, but the watershed was developed extensively; thus, the control and monitoring of water should be strengthened to mitigate the trend changes.

**Figure 5:**
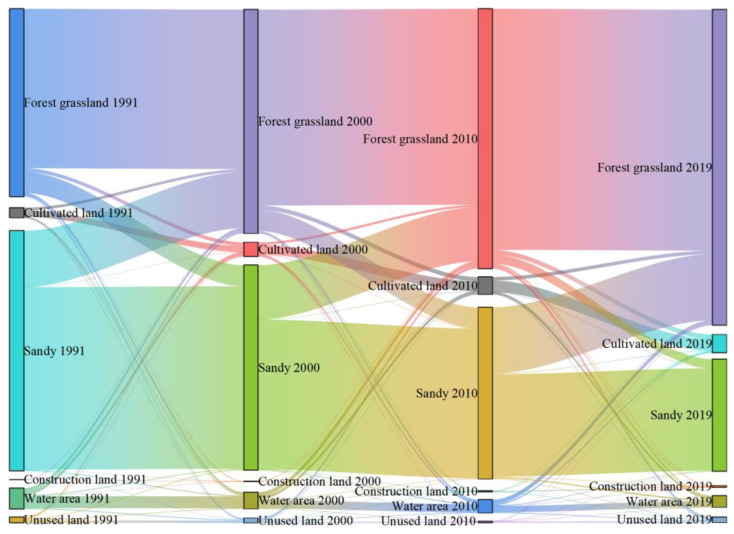
Land-use transfer in various periods along the Yellow River section of the Hobq Desert.

Land-use transfer changes along the Yellow River section of the Hobq Desert differed somewhat in several periods (Figure 5). During the first period, sandy land was most transferred, and the transformation of forest grassland to other land types followed, mainly to cultivated, water, and construction land. Furthermore, 44.58% of the transferred area from the water area was converted to cultivated land at that time. In the second period, construction land was exploited effectively, and the majority of shift type was forested grassland, with 67.84% of the total transferred area. There was a significant expansion in cultivated land, with the primary transfer being forest grassland. During the final period, sandy land always occupies the largest area transferred out, of which 14.98 km^2^ is developed as construction land due to urban expansion. Comparatively to the previous two phases, the amount and direction of land transfers increased in each type.

### 3.2. Landscape Pattern

**Figure 6:**
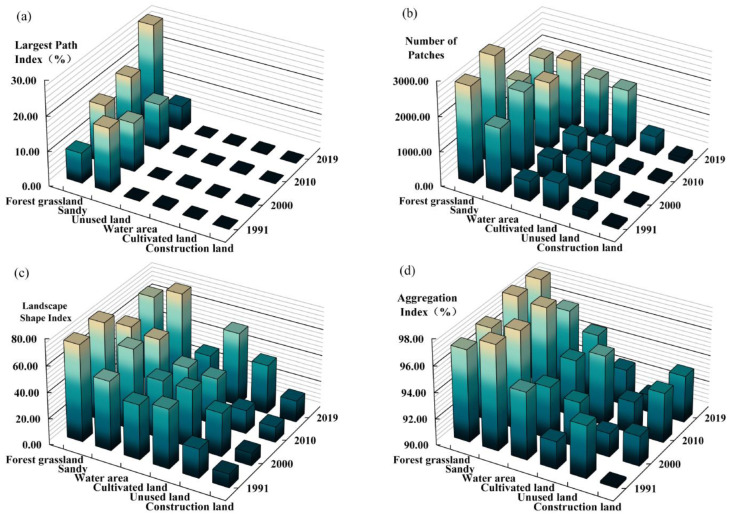
Changes in the landscape indexes at the class scale along the Yellow River section of the Hobq Desert from 1991 to 2019. (**a**) Largest Path Index. (**b**) Number of patches. (**c**) Landscape Shape Index. (**d**) Aggregation Index.

In this study, six types of land-use were analyzed at the class scale to determine the landscape patterns (Figure 6). The LPI describes the level of landscape dominance. It was found that the LPI of forest grassland was the largest in 2019, by 28.55%, while the sandy land decreased from 18.29% to 7.02%; this change shows that the replacement between landscape types coincides with the effect of sand control and management in the Hobq Desert. The NP is an index used to describe the heterogeneity of the landscape spatial pattern. The results showed that the NP of forest grasslands significantly exceeded that of other types between 1991 and 2019. Since 2010, the NP of the water area and cultivated land has risen markedly, and the landscape heterogeneity was obvious. The LSI reflects the complexity of the patch shape. Among them, forested grassland covers a large and stable area, and the overall change is not evident. On the contrary, the building land is subject to intense anthropogenic activities, the integrity of the landscape and the clusters are vulnerable and damaged, and the patch shape indexes are complicated. Additionally, a large amount of desert land has been reclaimed for agricultural production on the southern edge, increasing the shape index of cultivated patches. The AI shows the degree of aggregation of different land classes. The result indicates high AI levels in forest grassland and sandy areas, primarily due to the abundance of forest grassland and sandy areas and a smooth connection with the water area, while the regional landscape influences the unused land to a greater extent, and the landscape fragmentation is gradually broken up.

**Figure 7:**
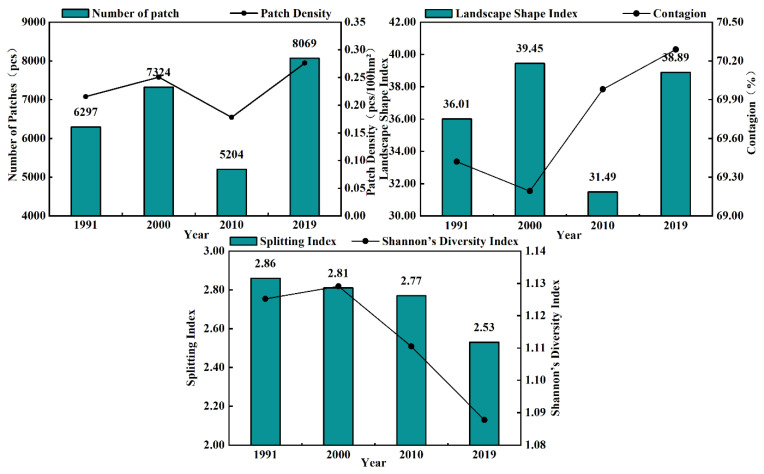
Changes in the landscape indexes (NP, PD, LSI, CONTAG, SPLIT, and SHDI) on the landscape scale along the Yellow River section of the Hobq Desert from 1991 to 2019.

From the general landscape-level changes in the desert region (Figure 7), both NP and PD showed “N” type fluctuations during 1991–2019. In general, this shows that the study area has seen a certain degree of expansion in landscape types and an increase in overall landscape fragmentation, where the NP and PD decreased from 2000 to 2010, mainly because environmental protection policies had been implemented. According to the patch shape index (LSI), the minimum value in 2010 was 31.49. The highest value at the study end was 38.89, reflecting that the landscape was disturbed by anthropogenic transformation in the study area. The edge shape became more complicated, and CONTAG increased significantly during this time, from 69.19% to 70.29%, indicating that patches of different landscape types gradually integrated through the ecological restoration process and landscape connectivity improved. In addition, the decrease in SPLIT and SHDI suggests that numerous small patches in the study area merged, causing a further homogenization of the landscape pattern.

### 3.3. Habitat Quality

#### 3.3.1. Temporal Changes in Habitat Quality

**Figure 8:**
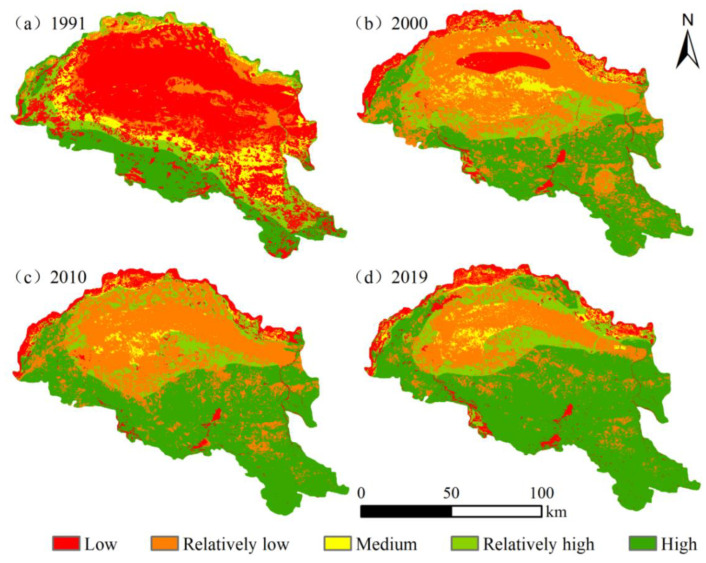
Spatial variation in habitat quality along the Yellow River section of the Hobq Desert from 1991 to 2019.

The InVEST model was used to generate habitat quality patterns over four time periods in 2019, 2010, 2000, and 1991 (Figure 8). We categorized them into five classes using the equal spacing method in ArcGIS: low (0~0.2), relatively low (0.2~0.4), medium (0.4~0.6), relatively high (0.6~0.8), and high (0.8~1). Spatially, the elevation of habitat quality along the Yellow River section of the Hobq Desert has a certain regularity. The overall pattern is high in the south and low in the north, high in the east and west, and down in the middle. Among them, low-grade and relatively low-grade habitat quality were distributed in the core desert area and around the Yellow River basin; the medium habitat quality was largely composed of the southern cultivated areas and a few forest grassland areas; high-grade and relatively high-grade habitat quality were mostly spread in the south-eastern area near to extensive forest grassland; and the plaque size and density expanded annually. There was significant variation in each habitat quality class area, and a bipolar approach was evident.

From a period perspective, it was the lower grade where habitat quality varied the clearest in the previous period, in which low-grade habitat quality decreased from 54.16% in 1991 to 10.92% in 2000, and relatively low-grade increased from 9.89% in 1991 to 41.09% in 2000 (Table 6). The habitat quality index is more steady over the medium term (2000–2010). From 2010 to 2019, there was a significant change in the quality of lower-rated habitats, with the proportion of area decreasing by 12.46%. From the overall analysis of the region, the average habitat quality in 1991, 2000, 2010, and 2019 was 0.3565, 0.5108, 0.5879, and 0.6482, respectively; the general habitat value shows a trend increase and the management of desert ecosystems was effective.

#### 3.3.2. Spatial Variation in Habitat Quality

**Figure 9:**
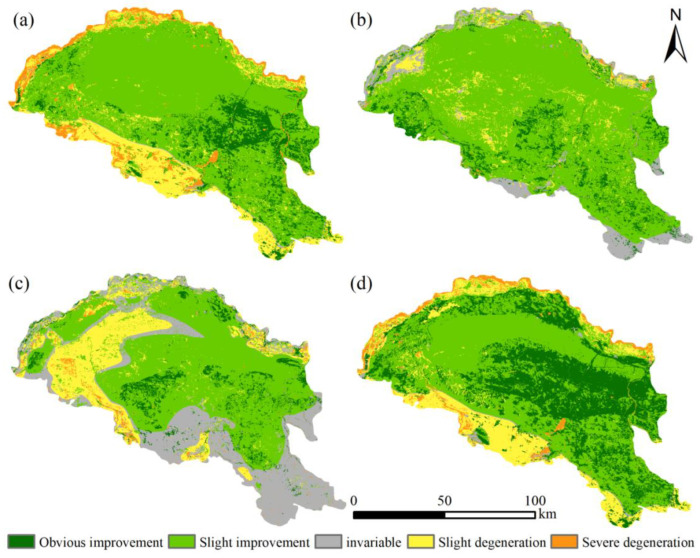
Spatial variation in habitat quality. (**a**) 1991–2000. (**b**) 2000–2010. (**c**) 2010–2019. (**d**) 1991–2019.

Further analysis of habitat quality variations along the Yellow River section of the Hobq Desert was performed by superimposing habitat quality classes. According to the magnitude of habitat quality changes, the following divisions have been made (Figure 9): habitat quality obvious improvement (>0.5), habitat quality slight improvement (0~0.5), habitat quality invariable (0.0), habitat quality slight degeneration (−0.5~0), and habitat quality severe degeneration (<−0.5).

From 1991 to 2000, habitat quality mainly increased, with only 0.65% of the stabilized area and frequent conversion among land types (Table 7). The slight improvement habitat quality was located in the center of the desert in a large distribution, accounting for 8509.13 km^2^; the obvious improvement habitat quality area was 1603.54 km^2^, showing a more fragmented distribution and showing it was affected by more obvious anthropogenic disturbance. There was a slight degeneration in habitat quality in 2043.77 km^2^, mainly near water areas and construction sites. As for 2000–2010, the habitat quality invariable areas were dramatically raised and distributed along the desert’s southern edge structure, the habitat quality slight improvement and slight degeneration areas were intertwined and closely linked, and landscape heterogeneity became enhanced. The total area of change from 2010 to 2019 amounts to 9547.37 km^2^, a decrease of 2279.05 km^2^ compared to the previous period. The habitat quality slight degeneration was concentrated in the northwestern part of the desert and increased significantly, reaching 2238.56 km^2^. In general, while the Hobq Desert developed the return of cultivated land to forest and grass in 1991–2019 within the ecologically vulnerable regions, it also emphasized ecological protection and vegetation recovery in the areas that had already returned to cultivated land. Thus, the habitat quality has become increasingly better. Nevertheless, expanding construction land and regulating farmland reduced habitat quality in some areas.

#### 3.3.3. Factors Influencing the Spatial Variation in Habitat Quality

**Figure 10:**
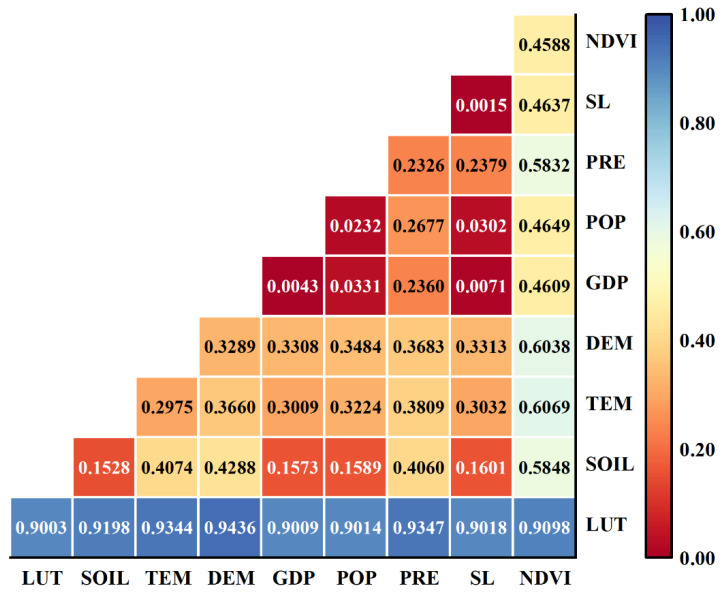
Interaction of influencing factors of spatial differentiation characteristics of habitat quality.

We used geographic detectors to analyze the factors causing spatial changes in habitat quality. It found that land-use type has a significantly higher determining power on habitat quality than other factors, with 0.9003, which is the major factor influencing spatial variation in habitat quality, followed by NDVI and DEM at 0.4588 and 0.3289, respectively, while GDP and population density also have little influence, indicating that natural factors have a greater impact than socioeconomic ones (Table 8). From the interaction of impact factors, it appears that different factors contribute to different levels of effectiveness: land-use type∩DEM (0.9436), land-use type∩precipitation (0.9347), and land-use type∩temperature (0.9322) were more strongly determined interactively, while NDVI∩temperature (0.6069), NDVI∩elevation (0.6038), and NDVI∩precipitation (0.5832) showed the following interaction determinants; slope∩GDP (0.0071) had the least interaction determinant and almost no effect on habitat quality. The interaction determinants between the two factors were higher than the determinants of individual factors in Figure 10, which means that the spatial differentiation pattern of habitat quality was driven by multiple factors combined. Among them, land-use type interacts most significantly with other factors since the land-use type determines habitat quality. This pattern greatly influences the spatial characteristics of habitat quality. The power to assess habitat quality by socioeconomic factors was minor; however, after interacting with natural factors, the ability to determine was increased, and the interaction intensity of the GDP factor arrived at 0.9009, reflecting the extent to which natural factors enhanced the influence of socioeconomic factors on habitat quality.

### 3.4. Prediction of Land Use and Habitat Quality (2030)

**Figure 11:**
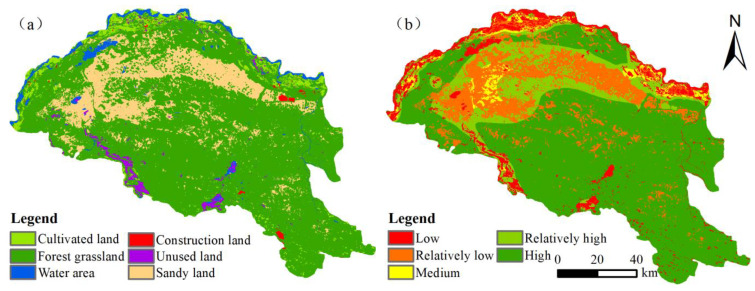
Spatial projections of land use and habitat quality along the Yellow River section of the Hobq Desert in 2030. (**a**) Land-use distribution. (**b**) Habitat quality.

To verify the prediction’s accuracy, we entered the actual data of 2019 with the forecasted land-use change result into the validation module of PLUS. We obtained a kappa coefficient of 0.69 and an FOM coefficient of 0.43, which indicated that the forecasting model had an optimistic prediction performance. Based on changes in the probability matrix of shifts for each land-use type in 2000–2010, projected land-use patterns are unaffected by policy adjustments through to 2030 (Figure 11). Table 9 shows a continuous increase in cultivated land, forest grassland, and construction land, and a decrease in sandy land. Among all types, the change in cultivated land is the steadiest, the evolution of unused land is the sharpest, and the rate of change in the unused land is 36.75% in 2019–2030.

Moreover, as shown in Table 10, the evolution pattern of habitat quality in 2019–2030 is the same as that in 2010–2019, with a moderate increase in the overall habitat quality score and an increase in the area with lower habitat quality in some areas, with a total habitat quality score of 0.6876 in 2030, increasing by 6.08% compared to 2019. The area of landscapes with low habitat quality continues to decrease, accounting for a total of 8.43% of the overall size, which is 23.99% less than in 2019, and the regions with a low habitat quality level are usually construction land, gathered in the southern edge of the desert cultivated land and around rivers. As a whole, the development trend for habitat quality improved significantly, with an increase in areas with high and invariable habitat quality. Compared to construction land and unused land, which have poor habitat suitability, forest grassland with appropriate habitat suitability has seen steady growth.

## 4. Discussion

### 4.1. Reasons for Land-Use Change

This study analyzed detailed monitoring of the spatio-temporal variations in land use along the Yellow River section of the Hobq Desert from 1991 to 2019. In each study area region, natural and human factors have combined to influence land use dynamically (Figure 5). The extent of sandy land and water areas decreased, and the size of cultivated land, forest grassland, unused land, and construction land increased; vegetation recovery is generally positive (Table 4).

Geographically, the overall land-use type displayed a trend of expansion from south to north and from outside to inside (Figure 4); these results are consistent with previous studies [47,48]. This spatial pattern shows that the external manifestation of the interaction caused anthropogenic activities and environmental change processes and the consequence was the interaction between desertification and oasis processes. Because of the increase in surface vegetation cover, desert detrital material is less eroded and transported, leading to the relative reduction in desert-spreading provenance [49].

From the natural factors, temperature and precipitation have a powerful limiting influence on the distribution of land use over long-term sequential scales [5]. The Hobq Desert is an ecologically fragile region in China, where suitable temperature and sufficient precipitation are necessary conditions for developing desert oasis vegetation [32]. The climate conditions improving in the desert in recent years might have positively influenced scrub and grassland growth, contributing to the expansion in vegetation cover [50]. Additionally, socio-economic development affects the structure and adjustment of land use. Based on the Ordos Statistical Yearbook, it can be found that the total population of Hangjin Banner in 1991 was 131.8 thousand, and the total population in 2019 was 143.7 thousand. Since 2001, when Ordos was dissolved as a municipality, it has become the fastest-growing region in the Inner Mongolia Autonomous Region. The GDP, primary, secondary, and tertiary industry output values of Hanjin Banner show a linear upward trend, the total value of GDP has increased 81.48 times, and illegal land reclamation, the overuse of land brought about by population growth, and a rapidly developing economy were the main drivers of the desert’s increase in cultivated land and construction land. Moreover, an effective ecological policy is imperative to reducing sandy land growth in the Hobq Desert and promoting restoration. The adoption of the grazing ban and grazing rest policy in Ordos, coupled with the implementation of natural forest protection, the “Three North” shelter forest development, and the conversion of farmland to forest environment restoration projects have brought a positive result to the vegetation and promoted urbanization in the study region; it is crucial to reverse the desertification along the Yellow River section of the Hobq Desert [51].

### 4.2. Impact of Land-Use Change on Habitat Quality

The findings suggest that land-use change was not ignored as a factor affecting regional habitat quality. It has a different impact on local economic development and ecological conservation in each region. Thus, analyzing the land-use effect on habitat quality is crucial for planning regional land use and developing ecologically sustainable communities. Land-use type drives landscape pattern changes that impact regional ecosystems’ structure and function [52]. The Hobq Desert along the Yellow River section comprises forest grassland, sandy land, cultivated land, and water area. This paper interprets the landscape pattern index changes in each land-use type, and these results suggest that the landscapes with forest grassland dominance increase, the leading type in sandy landscapes decreases, the patches of cultivated landscapes show a complex trend, and the distribution in construction and unused landscapes scatters with time (Figure 6). In summary, the ecological restoration process was sophisticated and regionally diverse in the Hobq Desert, with significant local land-use changes as the dryland economy keeps developing.

According to the InVEST-PLUS model, habitat quality has improved in the area. The overall pattern is high in the south and low in the north, high in the east and west, and down in the middle; this is evidenced by the shift from areas with lower habitat quality ratings to regions with relatively high and high habitat quality ratings (Figure 8). Areas of low habitat were located mostly near the Yellow River on the northern edge of the desert, with a complex structure of land-use types, including cultivated land, construction land, and water area, which were intensively used. In contrast, regions of high habitat quality were distributed on a large scale in forest grassland with a high adaptation of vegetation adaptability in the south–central desert. Moreover, in order to stop the desert from invading the Yellow River or expanding towards the south, people built a combination system of trees, shrubs, and grasses on the north and south edges of the Hobq Desert, which severely degraded the ecology and conditions meaning agricultural and livestock production were not available [53]. In addition, the Hobq Desert three-dimensional photovoltaic sand treatment model of energy treatment and transmission of green electricity is being extended to the western desert region, as the specific ecologically fragile area within the Yellow River basin. The spatial distribution of land-use patterns and the dynamic evolution of habitat quality in the Hobq Desert is related to optimizing land-use structure and effectively managing the ecological environment, which has great significance in the coordination of high-quality regional development and ecological civilization construction. In conjunction with the outline of the Plan for Ecological Protection and Quality Development of the Yellow River basin, the Hobq Desert region should proceed to improve the management system of the Yellow River basin, seriously implement ecological restoration, and continue to promote the following projects: repair destroyed habitats, facilitate the spatially coordinated development of land, and maintain habitat quality and regional environmental security.

### 4.3. Limitations and Uncertainties

In this paper, we analyzed land use and habitat quality changes along the Yellow River section of the Hobq Desert from 1991–2019, while simulating future development conditions in 2030. Firstly, we used the InVEST model to analyze changes in habitat quality conditions at intervals of about ten years based on the original land-use data of the last thirty years (1991, 2000, 2010, and 2019). After that, we selected 12 drivers from socio-economic and natural factors and applied the PLUS model to project the distribution of land-use types in 2030 based on 2019 land-use data. Liang et al. [30] developed the PLUS model which was continuously improved upon the FLUS model, where land-use types are available at patch scales for more precision. As a result, it has been demonstrated that the PLUS model simulates more accurately and provides landscape patterns that correlate better with reality than other CA-based models. Finally, we used the simulated 2030 land-use-type data as the basis for the analysis of habitat quality using the InVEST model to provide decision support for the sustainable development of the Hobq Desert resources. At the same time, we used the geodetector model to reveal the drivers of spatial variation in habitat quality, overcoming the previous inability to capture spatial dependence and spillover effects using linear model analysis. The results provide an excellent reference for ecological environment protection and regional sustainable development in the Hobq Desert.

However, despite the fact that the habitat quality module of the InVEST has relatively matured, the majority of the research has focused on the operation manual and related studies, which carry certain subjectivity. The habitat quality module focuses on human-influenced threat factors, ignoring internal threats to a limited extent [54]. Furthermore, this paper couples the PLUS model with the InVEST model to quantitatively assess future land-use change and its impact on habitat quality to compensate for the shortcomings of a single model. Meanwhile, the InVEST-PLUS model also has limitations. Due to the differences in the understanding or knowledge level of land use and habitat quality in different regions and the inconsistency in the selection of indicators, there are uncertainties in the estimation results, so the continuous monitoring of land use in the field should be strengthened in future studies to verify the rationality of the model in a timely manner. In this study, with the limitations of data, we only used the InVEST-PLUS coupled model to predict the land-use changes in the inertial development conditions without adding a variety of restricting factors for multi-scenario evaluation analysis, which has problems related to exploring the optimal parameter settings and model prediction accuracy. The habitat quality under multiple ecological functions needs to improve and be examined in depth for accurate regional decision-making.

## 5. Conclusions

At present, the northwest arid area is witnessing rapid development with land use in a transitional state. This paper takes the Hobq Desert along the Yellow River section as the study area, which is a key region for sand control and sand management in the Yellow River, and a large number of ecological restoration measures and desert treatment projects were carried out during the study period. Desertification control is related to people’s living environments in desert areas, global climate change, and sustainable development of the economy and society. Therefore, monitoring the dynamics of land use and environments over long time series is of scientific importance to understand the regional response to global change. In this paper, we detailed the land-use and landscape pattern changes in the last three decades, simulated future land-use development, and explored the reasons for the habitat quality change patterns and spatial heterogeneity. The conclusions are as follows:

(1) From 1991 to 2019, the transition of all land types was frequently in the region studied; numerous areas of sandy land were turned into forest grassland, and the percentage of cultivated land and construction land continued to increase, whereas the water area decreased continuously, and the unused land decreased first and improved later. Among them, the construction land grew fastest in 2000–2010; the unused land expanded sharply in 2010–2019, and the highest comprehensive land-use dynamics were in the final period, with the most dramatic degree of land-use change.
(2) As a result of the landscape indices, land use landscape fragmentation has increased, landscape connectivity has become better, and the overall landscape type has developed in a balanced manner. In terms of plaque type, the landscapes with forest grassland dominance increased, the leading variety in sandy landscapes decreased, the patches of cultivated landscapes showed complex trends, and the distribution in construction and unused landscapes scattered with time.
(3) Habitat quality in the Hobq Desert from 1991 to 2019 shows positive development, evidenced by the shift from regions with relatively low and low habitat quality ratings to those with relatively high and high habitat quality ratings. In terms of spatial distribution, the habitat quality has a certain regularity, and the overall pattern is high in the south, low in the north, high in the east and west, and low in the middle.
(4) In terms of the prediction, land-use change in the study area from 2019 to 2030 will basically be the same as in the previous period, but the speed will generally decrease. The concentration of unused land in the northwest corner of the desert will increase significantly. Meanwhile, the variation in habitat quality intensity will fall, the areas with invariable-grade habitat quality will increase, and the spatial distribution pattern will change slightly.

## Figures and Tables

**Table 1:** Landscape indices formula and ecological meaning.

Index	Expressions	Unit	Applied Scale	Ecological Meaning
patch density (PD)	PD = N/A	Pcs/hm^2^	landscape	Describes the degree of landscape fragmentation
number of patches (NP)	NP = N	Pcs	patch class/landscape	Describes the total number of landscapes
landscape shape index (LSI)	$LSI=\frac{e^{i}}{\min e^{i}}$	None	patch class/landscape	Describes the shape of landscapes
largest patch index (LPI)	$LPI=\frac{Max(a_{1},\ldots ,a_{n})}{A_{i}}\times 100$	%	patch class/landscape	Describes the dominant landscape
splitting index (SPLIT)	$SPLIT=\frac{A^{2}}{\sum_{j=1}^{n}{a_{ij}}^{2}}$	None	landscape	Describes the separation level of landscapes
Shannon’s diversity index (SHDI)	$SHDI=-\sum_{i=1}^{m}p_{i}(\ln p_{i})$	None	landscape	Describes the diversity of landscapes
aggregation index (AI)	$AI=\left[\sum_{i=1}^{m}(\frac{g_{ii}}{\max g_{ii}})p_{i}\right]\times 100$	%	patch class/landscape	Describes the degree of landscape aggregation
contagion index (CONTAG)	$CONTAG=\left[1+\frac{\sum_{i=1}^{m}\sum_{k=1}^{m}\left[(P_{i})\left(\frac{g_{ik}}{\sum_{k=1}^{m}g_{ik}}\right)\right]\left[\ln\left(P_{i}\right)\left(\frac{g_{ik}}{\sum_{k=1}^{m}g_{ik}}\right)\right]}{2\ln(m)}\right](100)$	%	landscape	Describes landscape connectivity

In the table, *e^i^* is the circumference of a certain plaque type; min *e^i^* is the square perimeter of the total area of the patches in class *i*; *A* represents landscape area; *a* represents patch area; *m* is the total number of the landscape; and *g* represents the number of pixels between different patches of the same patch type.

**Table 2:** Maximum influence distance and weight of threat factors.

Threat Factors	Maximum Effective Distance (km)	Weight	DECAY
Cultivated land	4	0.6	linear
Construction land	8	0.9	exponential
Unused land	6	0.5	linear
Sandy land	6	0.75	exponential

**Table 3:** Sensitivity of different land-use types to habitat threat factors.

Land-Use Types	Habitat Suitability	Threat Factors			
		Cultivated Land	Construction Land	Unused Land	Sandy Land
Sandy land	0.1	0.1	0.3	0.3	0.1
Forest grassland	1	0.7	0.6	0.6	0.7
Water	0.9	0.6	0.2	0.4	0.3
Unused land	0.1	0.1	0.3	0.1	0.6
Cultivated land	0.4	0.3	0.5	0.4	0.3
Construction land	0	0.1	0.3	0.2	0

**Table 4:** The area of land-use change in various periods along the Yellow River section of the Hobq Desert.

Year	Cultivated Land		Forest Grassland		Water Area		Construction Land		Unused Land		Sandy Land	
	Area (km^2^)	%	Area (km^2^)	%	Area (km^2^)	%	Area (km^2^)	%	Area (km^2^)	%	Area (km^2^)	%
1991	284.61	2.19	5254.91	40.39	583.42	4.48	8.45	0.06	162.08	1.25	6718.67	51.63
2000	394.73	3.03	6260.96	48.12	467.77	3.6	11.42	0.09	139.3	1.07	5737.09	44.09
2010	489.14	3.76	7267.08	55.85	376.69	2.9	27.28	0.21	44.55	0.34	4806.61	36.94
2019	511.67	3.93	8827.41	67.84	322.67	2.48	50.76	0.39	158.38	1.22	3140.46	24.14

**Table 5:** The land-use dynamics in various periods along the Yellow River section of the Hobq Desert.

Land-Use Type	Change in Area (km^2^)				Land-Use Dynamics				Comprehensive Land-Use Dynamics			
	1991–2000	2000–2010	2010–2019	1991–2019	1991–2000	2000–2010	2010–2019	1991–2019	1991–2000	2000–2010	2010–2019	1991–2019
Cultivated land	110.12	94.41	22.53	227.06	4.3	2.39	0.46	4.93	1.22	1.08	1.69	0.68
Forest grassland	1006.05	1006.12	1560.33	3572.5	2.13	1.61	2.15	4.5				
Water area	−115.65	−91.08	−54.09	−260.82	−2.2	−1.95	−1.44	−8.98				
Construction land	2.97	15.86	23.48	42.31	3.91	13.89	8.61	9.26				
Unused land	−22.78	−94.75	113.83	−3.7	−1.57	−6.8	25.55	−0.26				
Sandy land	−981.58	−930.48	−1666.15	−3578.21	−1.62	−1.62	−3.47	−12.66				

**Table 6:** Proportion of each habitat quality level along the Yellow River section of Hobq from 1991 to 2019.

Grade	1991		2000		2010		2019	
	Area	Percentage	Area	Percentage	Area	Percentage	Area	Percentage
Low	7046.91	54.16	1420.42	10.92	912.78	7.02	996.03	7.66
Relatively low	1287.31	9.89	5346.16	41.09	4841.59	37.21	3220.80	24.75
Medium	1068.57	8.21	764.41	5.87	288.84	2.22	395.16	3.04
Relatively high	1317.91	10.13	1299.37	9.99	1229.24	9.45	1462.02	11.24
High	2290.10	17.60	4180.95	32.13	5738.84	44.11	6937.29	53.32

**Table 7:** Area and proportion of habitat quality grades along the Yellow River section of the Hobq Desert in 1991–2019.

Changes in Habitat Quality	1991–2000		2000–2010		2010–2019		1991–2019	
	Area	%	Area	%	Area	%	Area	%
Obvious improvement	1603.54	12.32	1161.47	8.93	865.90	6.66	4255.01	32.71
Slight improvement	8509.13	65.40	9690.60	74.49	6194.69	47.62	6129.32	47.12
Invariable	84.32	0.65	1182.87	9.09	3461.92	26.61	142.22	1.09
Slight degeneration	2043.77	15.71	892.42	6.86	2238.56	17.21	1946.91	14.97
Severe degeneration	770.03	5.92	81.98	0.63	248.24	1.91	535.74	4.12

**Table 8:** Complex drivers of habitat quality change.

Influence Factors	Land-Use Type (LUT)	Soil Type (SOIL)	Temperature (TEM)	Elevation (DEM)	GDP	Population Density (POP)	Precipitation (PRE)	Slope (SL)	NDVI
*q* value	0.9003	0.1528	0.2975	0.3289	0.0043	0.0232	0.2326	0.0015	0.4588

**Table 9:** Land-use forecast along the Yellow River section of the Hobq Desert in 2030.

Land-Use Type	Area (km^2^)		Change in Area (km^2^)	Change Rate (%)
	2019	2030		
Cultivated land	511.67	522.35	10.68	2.09
Forest grassland	8827.41	9719.40	891.99	10.10
Water area	322.67	355.98	33.31	10.32
Construction land	50.76	55.72	4.96	9.77
Unused land	158.38	216.59	58.21	36.75
Sandy	3140.46	2139.22	−1001.24	−31.88

**Table 10:** Habitat quality forecast along the Yellow River section of the Hobq Desert in 2030.

Grade	2019		2030		2019–2030	
	Area	Percentage	Area	Percentage	Area	Percentage
Low	996.03	7.66	355.99	2.74	−640.04	−4.92
Relatively low	3220.80	24.75	739.69	5.69	−2481.11	−19.07
Medium	395.16	3.04	2229.41	17.14	1834.25	14.10
Relatively high	1462.02	11.24	320.71	2.47	−1141.31	−8.77
High	6937.29	53.32	9363.58	71.98	2426.29	18.66

## Data Availability

Not applicable.
